# Prediction of the occurrence of leprosy reactions based on Bayesian networks

**DOI:** 10.3389/fmed.2023.1233220

**Published:** 2023-07-26

**Authors:** Rafael Saraiva de Andrade Rodrigues, Eduardo Ferreira José Heise, Luis Felipe Hartmann, Guilherme Eduardo Rocha, Marcia Olandoski, Mariane Martins de Araújo Stefani, Ana Carla Pereira Latini, Cleverson Teixeira Soares, Andrea Belone, Patrícia Sammarco Rosa, Maria Araci de Andrade Pontes, Heitor de Sá Gonçalves, Rossilene Cruz, Maria Lúcia Fernandes Penna, Deborah Ribeiro Carvalho, Vinicius Medeiros Fava, Samira Bührer-Sékula, Gerson Oliveira Penna, Claudia Maria Cabral Moro, Julio Cesar Nievola, Marcelo Távora Mira

**Affiliations:** ^1^School of Medicine and Life Sciences, Graduate Program in Health Sciences, Pontifícia Universidade Católica do Paraná – PUCPR, Curitiba, Paraná, Brazil; ^2^Graduate Program in Health Technology, PUCPR, Curitiba, Paraná, Brazil; ^3^Tropical Pathology and Public Health Institute, Federal University of Goiás, Goiania, Brazil; ^4^Instituto Lauro de Souza Lima, Bauru, São Paulo, Brazil; ^5^Dona Libânia Dermatology Centre, Ceará, Brazil; ^6^Tropical Dermatology and Venerology Alfredo da Matta Foundation, Amazonas, Brazil; ^7^Epidemiology and Biostatistics Department, Federal University Fluminense, Rio de Janeiro, Brazil; ^8^Program in Infectious Diseases and Immunity in Global Health, Research Institute of the McGill University Health Centre, and The McGill International TB Centre, Departments of Human Genetics and Medicine, McGill University, Montreal, QC, Canada; ^9^Tropical Medicine Centre, University of Brasília, and Fiocruz School of Government – Brasilia, Brasília, Brazil; ^10^Graduate Program in Informatics, PUCPR, Curitiba, Paraná, Brazil; ^11^Pharmacy Program, School of Health and Biosciences, PUCPR, Curitiba, Paraná, Brazil

**Keywords:** leprosy, leprosy reactions, risk, Bayesian networks, artificial intelligence

## Abstract

**Introduction:**

Leprosy reactions (LR) are severe episodes of intense activation of the host inflammatory response of uncertain etiology, today the leading cause of permanent nerve damage in leprosy patients. Several genetic and non-genetic risk factors for LR have been described; however, there are limited attempts to combine this information to estimate the risk of a leprosy patient developing LR. Here we present an artificial intelligence (AI)-based system that can assess LR risk using clinical, demographic, and genetic data.

**Methods:**

The study includes four datasets from different regions of Brazil, totalizing 1,450 leprosy patients followed prospectively for at least 2 years to assess the occurrence of LR. Data mining using WEKA software was performed following a two-step protocol to select the variables included in the AI system, based on Bayesian Networks, and developed using the NETICA software.

**Results:**

Analysis of the complete database resulted in a system able to estimate LR risk with 82.7% accuracy, 79.3% sensitivity, and 86.2% specificity. When using only databases for which host genetic information associated with LR was included, the performance increased to 87.7% accuracy, 85.7% sensitivity, and 89.4% specificity.

**Conclusion:**

We produced an easy-to-use, online, free-access system that identifies leprosy patients at risk of developing LR. Risk assessment of LR for individual patients may detect candidates for close monitoring, with a potentially positive impact on the prevention of permanent disabilities, the quality of life of the patients, and upon leprosy control programs.

## 1. Introduction

Leprosy is a chronic, disabling infectious disease caused by *Mycobacterium leprae* (*M. leprae*) (1) that affected 141,000 new individuals worldwide in 2021 – a number likely to be underestimated due to potential sub-notification caused by the COVID-19 pandemic – with most cases concentrated in India and Brazil (2). In the classical Ridley & Jopling (R&J) classification system, tuberculoid (TT) and lepromatous (LL) leprosy occupy opposite ends of a continuous disease spectrum that includes three borderline forms (BT, BB, and BL) (3). The TT + BT and BB + BL + LL cases roughly correspond to paucibacillary (PB) and multibacillary (MB) leprosy, according to the treatment-oriented World Health Organization (WHO) classification scheme, respectively (2, 4, 5). Today, it is widely accepted that exposure to *M. leprae* is necessary but not sufficient for the development of leprosy; different sets of host gene variants mediate susceptibility to leprosy in three different stages (6): (i) controlling infection *per se*, that is, the disease regardless of its clinical presentation, (ii) defining the clinical form of disease after the infection is established, and (iii) outlining the risk of developing leprosy reactions (LR) (7, 8).

Leprosy reactions are characterized by an intense and sudden (re)activation of the host inflammatory response that may be diagnosed concomitantly with leprosy, during or even after treatment (9–12). Upon diagnosis, LR requires immediate medical attention to prevent irreversible nerve damage, motor disability, and permanent anatomical deformities. In 2021, 6.04% of newly detected leprosy cases worldwide presented grade-2 disabilities in the diagnosis (2), often due to LR. Cohort studies estimate that, during leprosy, 16 to 56% of the patients will develop irreversible nerve damage, again, mainly due to reactional episodes (13–16). Over the past years, advances in genetic research improved our understanding of the molecular basis of leprosy pathogenesis, and several host genetic variations have been implicated in the control of LR episodes (17–19).

There are two major types of LR of distinct clinical presentation: type-1 (T1R) and type-2 reaction (T2R). T1R affects 10–30% of leprosy patients and occurs primarily within, but not limited to, the first 2 years after leprosy diagnosis (20, 21). Known risk factors for T1R are (i) borderline clinical groups BT-BL (22); (ii) age of leprosy onset, with older individuals being at higher risk (23, 24); (iii) positive bacillary index (25); (iv) an increased number of lesions at leprosy diagnosis (26, 27); (v) detection of *M. leprae* DNA in biopsies of lesions (24); and (vi) genetic/genomic studies have identified an association between T1R and genes *TLR1* (28), *TLR2* (29), *TLR3* (30), *TLR7* (30), *TLR10* (30), *NRAMP1/SCLC11A1* (31), *VRD* (32), *NOD2* (33), *TNFSF15*/*TNFSF8* (34, 35), lncRNA *ENSG00000235140* (36), *LRRK2* (19), and *PRKN* (19).

Leprosy T2R mainly affects patients classified within the BB-LL range (13, 37). Patients presenting bacterial index higher than 4+ in skin smears are at increased risk for T2R (38, 39). There is a wide variation in the prevalence of T2R in different geographic and endemic settings. In Brazil, approximately 37% of BL and LL cases develop T2R, while in India, Nepal, and Thailand, the proportion is between 19–26% (40). A prospective study involving BL and LL patients from India followed for 11 years, showed that less than 10% of the individuals who developed T2R had a single episode, whereas 62% had chronic T2R (21). In Ethiopia, 63% of leprosy cases had more than one T2R episode, while 37% had a single event (41). Host genetics also seems to play a significant role in controlling the occurrence of T2R, and genes *C4B* (42), *TLR1* (43), *NRAMP1*/*SCLC11A1* (31), *NOD2* (33, 35), and *IL6* (12, 35) have been implicated as critical molecular players.

One of the challenges of translational medicine is to systematize the analysis of a large amount of patient data to predict a specific outcome. In addition, scientific results from basic research are often difficult to translate into daily medical practice. Artificial Intelligence (AI) methods seek to systematically address often large, complex data sets to provide a base for decision-making. Of particular interest in health care, Bayesian Networks (BN) are among the most successful techniques in processing and unraveling the relationship between a large number of variables, with risk estimation being the outcome (44).

A Bayesian Network (BN) is a graphical model of an outcome variable’s posterior conditional probability distribution based on evidence. It contains nodes that represent the random variables and links between pairs of nodes, which represent the causal relationship of these nodes, together with a conditional probability distribution in each node. From the definition, one can deduce that any joint probability distribution may be represented by a Bayesian network, which shows its modeling power: any deterministic model is a particular case of a probabilistic model, and any probabilistic model may be represented as a Bayesian network (45, 46).

Several BN-based systems have been created using medical data, developed for different purposes, and applied to several health conditions such as cardiovascular diseases, liver diseases, cancer and Alzheimer’s disease (47–57), including leprosy (44, 58–65). However, few initiatives aim to systematize a large amount of existing information of distinct nature to estimate the risk of the occurrence of a particular event. In the context of leprosy, creating a simple-to-use and flexible platform to predict the risk of LR based on patient data may help minimize the consequences of such aggressive events. Moreover, such a tool could improve leprosy control initiatives and public health systems. Here we present an AI system designed to predict the risk of a leprosy patient to develop LR using a complete or partial dataset of clinical, demographic, and host genetic data.

## 2. Materials and methods

**Figure 1:**
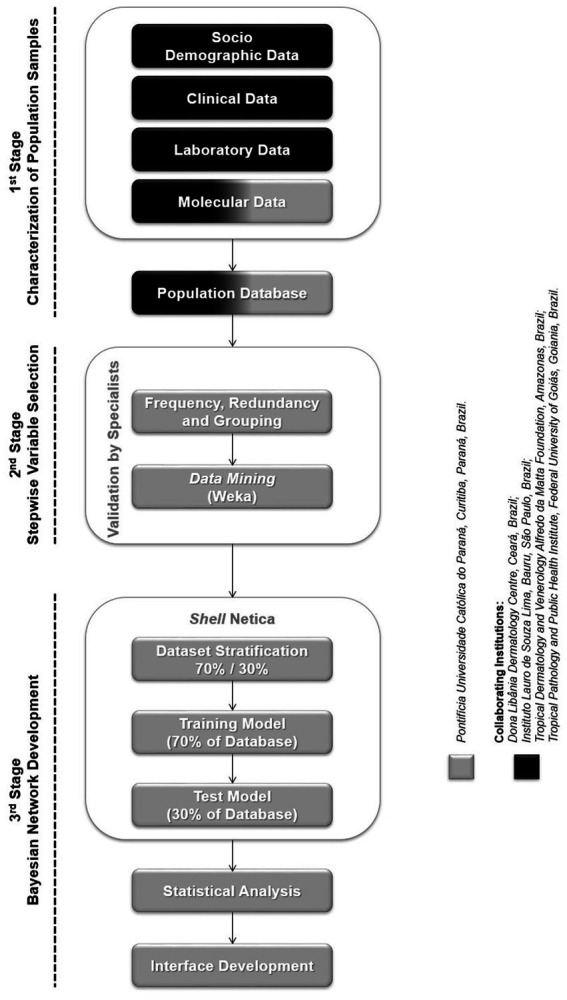
Flowchart: study design.

A flowchart summarizing the three stages of the study and the procedures described next is available in Figure 1.

### 2.1. Population samples

This study used four pre-existing data sets from previous research initiatives of different/independent designs and contexts. The first database included in the study consisted of 409 leprosy patients diagnosed at the Reference Center for Diagnosis and Therapy located in Goiania, central-western Brazil, between February 2006 and March 2008, originally used for the genetic study that identified an association between T2R and variants of the *IL6* gene. A complete description of the Goiania population has been published elsewhere (12). Later, the Goiania population was used for an expanded investigation involving a larger number of candidate genes that detected an association between T1R and variants of the gene *TNFSF8* (34). Finally, an association between T1R and lncRNA *ENSG00000235140* (36) and *LRRK2* (unpublished data) was also detected in the Goiania sample. Two additional databases comprised 533 patients recruited at the Dermatological Center Dona Libânia, Fortaleza, northeast Brazil, and 137 patients diagnosed with leprosy at the Fundação Alfredo da Matta, Manaus, north Brazil. Enrolment of these two population samples was performed under a single protocol of a clinical study described previously (66) and conducted by the Tropical Medicine Center of the University of Brasília between March 2007 and February 2012. Finally, a fourth database consisted of 371 patients diagnosed with leprosy at the Instituto Lauro de Souza Lima, Bauru, southeast Brazil, between March 2008 and January 2013, originally for a genetic study that detected an association between leprosy and variants of the *TLR1* (67) and *NOD2* (68) genes. For all databases, leprosy diagnosis/classification was defined after detailed dermatological and neurological examination by specialized leprologists, complemented by bacilloscopy and histopathology of skin lesions. All cases were classified following the R&J scheme (3). Patients were followed up for at least 2 years since diagnosis to monitor LR occurrence. Individuals who did not present LR at the initial diagnosis or during follow-up, were defined as non-reactional leprosy patients.

All patients were treated for leprosy according to WHO/MDT guidelines and for LR with the appropriate therapy. All subjects were evaluated for an extensive clinical, socioeconomic, and demographic information list.

### 2.2. Variable selection

The four databases included in this study were composed of clinical and laboratory parameters, most of them obtained for descriptive, epidemiological purposes unrelated to the occurrence of LR. Each one of the databases was subjected individually to a two-step, unbiased process aiming to identify those variables exerting the highest impact upon the risk of LR, thus, to be included in the system, as follows:

#### 2.2.1. Frequency, redundancy, and grouping

The first selection step consisted of removing variables with low frequency (less than 15%) of occurrence and/or mutually correlated (redundant), consequently capturing the same information. In the case of redundant variables, the most frequent was selected to capture the information of the set.

#### 2.2.2. Data mining

Data mining is one of the main stages of the knowledge extraction process from large databases, also known as KDD – Knowledge Discovery in the Databases (69). This AI method is defined as the process of discovering patterns in data to generate helpful information for the decision-making (70). WEKA (Waikato Environment for Knowledge Analysis) is an open-source program with a collection of algorithm implementations of various data mining techniques, such as pre-processing, classification, clustering, and visualization (71). This study used WEKA in the second variable selection step to identify those hierarchically important for LR occurrence in the population samples. The variables were selected using the C4.5 algorithm, which creates a decision tree and identifies the most relevant and non-redundant variants, thus reducing the number of attributes. The C4.5 selection is made according to the gain ratio, which is a normalization of the information gain, a parameter based on the entropy measure (originating from information theory) closely related to the maximum likelihood estimations (MLE) and usually used to make inferences about parameters of the underlying probability distribution from a given dataset (45, 72–75).

Four dermatologists/hansenologists with extensive experience in the area continuously validated the two-step variable selection through a qualitative process based on their experience in the field of leprosy diagnosis. These specialists were also involved in conducting system performance assessments, evaluating usability, and organizing the workflow for integrating data from the four databases. By leveraging the knowledge and expertise of specialists, clinical decision systems can be effectively validated and optimized for real-world clinical use (76). Criteria for selecting the specialists were; (i) holding MD/Ph.D. degrees in dermatology/hansenology; (ii) having more than 10 years of experience in leprosy diagnosis; (iii) being representative of regions of Brazil with different levels of leprosy endemicity.

Finally, two datasets contributed with genotypic information: Goiania for genes *IL6, TNFSF8*, *LRRK2*, and *ENSG00000235140* and Bauru for *TLR1* and *NOD2*, all previously studied in these population samples.

### 2.3. System development

The system was created as a BN using Shell NETICA (Norsys Software Corporation) (77) with a customized dynamic interface considering the number of variables in the database. The system was designed to operate with complete or partial information, which is of critical importance considering the translational bias of the proposal and the fact that several leprosy centers may not have access to all the information included, particularly the molecular genetic data. The system loads a spreadsheet in which columns and lines refer to the variables and records, respectively. Each variable (column) is related to one node of the BN. The variables comprise demographic, clinical, laboratory, and genetic data (markers). For each one of the databases, two groups were formed randomly to create the network: the test file, with 30% of patients, and the training file, with 70% of patients, both stored in an Excel file format.

The system’s performance was assessed by its accuracy, sensitivity, specificity, and negative and positive predictive values. The patient’s predicted outcome was defined by the class with higher risk, as estimated by the system. Predictive values were calculated using the prevalence of occurrence of reversal reactions observed for the studied population samples. The feature “importance” was also measured using the $F_{1}$ score, which is the harmonic mean between positive predictive value (PPV) and sensitivity. The $F_{1}$ score was calculated accordingly to the equation $F_{1}\;score=2*\frac{PPV*sensitivity}{PPV+sensitivity}$ using Python 3.7.9.

## 3. Results

Table 1 summarizes information on age, gender, and clinical form of leprosy according to the R&J classification system for T1R, T2R and non-reactional leprosy patients groups of all population samples. The mean age at diagnosis ranged from 40 to 59 years old, and males were consistently more frequent than females across all four population samples. Leprosy clinical form most frequently observed was BT (479, 33%) followed by BL (379, 26.1%), LL (346, 23.8%), BB (134, 9.2%), and TT (100, 6.9%). For the combined sample, 51% were non-reactional leprosy patients, 25.9 and 23.1% developed T1R or T2R, respectively. As expected, T1R was observed more often in BT + BB + BL cases, and T2R occurred more often in BL and LL individuals (Table 1).

**Table 1:** Distribution of sex, age at diagnosis, and clinical type of disease of leprosy-affected individuals with T1R, T2R, and non-reactional leprosy patients in each population sample.

	Patients, No. (%)														
	Goiania			Fortaleza			Manaus			Bauru			Combined		
Age, Years (Mean ± SD)	44.63 ± 16.67			45.15 ± 14.25			40.00 ± 15.39			59.00 ± 18.04			48.00 ± 17.29		
Sex															
Male	234 (57.1)			352 (66.0)			100 (72.9)			258 (69.5)			944 (65.1)		
Female	175 (42.9)			181 (34.0)			37 (27.1)			113 (30.5)			506 (34.9)		
Ridley&Jopling Classification	NRLP	T1R	T2R	NRLP	T1R	T2R	NRLP	T1R	T2R	NRLP	T1R	T2R	NRLP	T1R	T2R
TT	22	0	0	28	0	0	16	0	0	34	0	0	100	0	0
BT	124	79	0	164	24	0	36	4	0	18	30	0	342	137	0
BB	16	29	3	12	14	0	2	3	0	27	27	1	57	73	4
BL	26	46	8	47	71	66	12	28	10	12	20	33	97	165	117
LL	28	0	28	33	0	68	5	0	16	66	0	102	132	0	214
I	0	0	0	6	0	0	5	0	0	1	0	0	12	0	0
HI (Mean)	–	–	–	–	–	–	–	–	–	1.73	2.69	3.84	1.73	2.69	3.84
Proportion per Group	52.9	37.6	9.5	54.4	20.5	25.1	55.5	25.5	19.0	42.6	20.8	36.6	51.0	25.9	23.1
Total	409			533			137			371			1,450		

BB, borderline borderline; BL, borderline lepromatous; BT, borderline tuberculoid; HI, histological index; I, indeterminate leprosy; LL, lepromatous leprosy; NRLP, non-reactional leprosy patients; SD, standard deviation; TT, tuberculoid leprosy; T1R, type-1 reaction; T2R, type-2 reaction.

Our strategy for variable selection led to the inclusion of 34 demographic, clinical, laboratory, and genetic parameters (Supplementary Table S1) related to the occurrence of LR in the population samples (Table 2). Since the initial set of variables was not the same across the four datasets – thus, the variables selected by the two-step process and validated by the specialists were not necessarily the same – the prediction system was designed to include all variables selected in each population sample. Detailed information about the distribution of the included variables across the four different datasets is available in Supplementary Table S2.

**Table 2:** Demographical, clinical, laboratory, and genetic variables selected in the study.

Data	Variable information^a^
Socio-demographic	Sex
	Age group
	Ethnicity
Clinical	Multidrug therapy
	First signs and symptoms
	Ridley-Jopling classification
	Number of skin lesions
	Type of lesion
	Color of lesion
	Sensibility testing
Laboratory	Bacilloscopic index
	Histological index
	PGL-1
Genetic	*IL6* markers (4)
	*NOD2* marker (1)
	*TLR1* markers (2)
	*TNFSF8* markers (4)
	*ENSG00000235140*markers (4)
	*LRRK2* markers (3)
Family History	First degree^b^
	Second degree^c^
	Contact^d^

a Self-report in years since noticing the early signs and symptoms of leprosy.

b Father, mother, child, and sibs affected by leprosy.

c Cousins, nephews, uncles/aunts, grandparents, and grandchildren affected by leprosy.

d Close household contact affected by leprosy.

**Figure 2:**
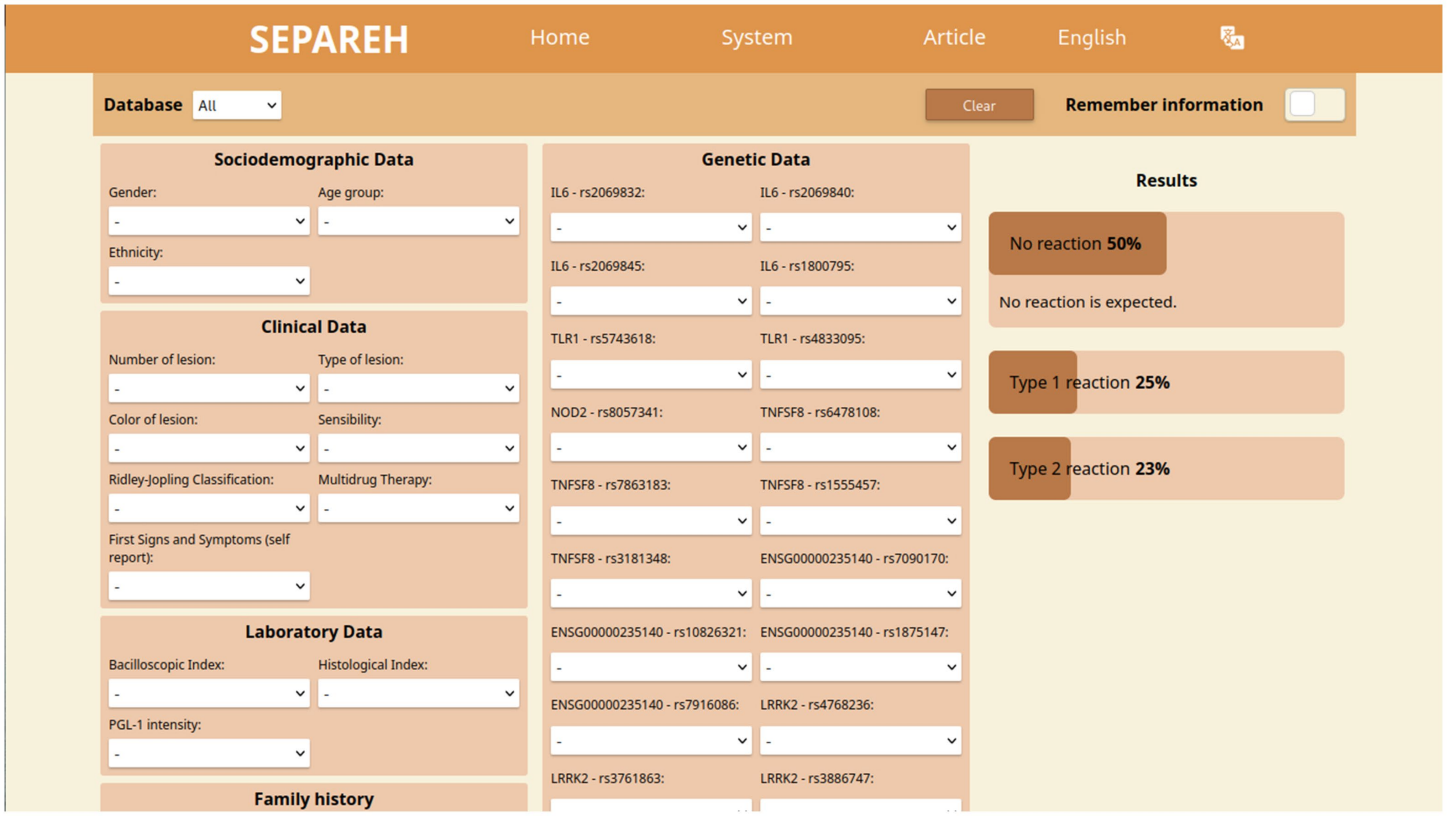
SEPAREH interface.

The risk-prediction system was developed to allow the use of each of the four databases individually as a reference, as well as to use a single, combined dataset, thus favoring customization and facilitating the inclusion of new data sets. The system – named SEPAREH (from Portuguese: *Sistema Especialista Para Avaliação de Risco de Estado Reacional em Hanseníase*; in English: Specialist System for Evaluation of Risk of Occurrence of Reactional States in Leprosy) is designed to present a friendly graphical user interface (Figure 2), which allows the primary care professional to use it intuitively. Variation of the patient’s risk of developing one of the two types of LR is shown in real time, as each available clinical and/or genetic information is included in the interface. The platform can be accessed for free at https://orfeu.ppgia.pucpr.br/separeh.^1^

The overall sensitivity and specificity of the system, as estimated using the combined dataset of 1,450 patients, was 79.3% (95% CI 73.9–84.7) and 86.2% (95% CI 81.6–90.8), respectively. Accuracy reached 82.7% (95% CI 79.2–86.3), and positive and negative predicted values were 85.1% (95% CI 80.2–90.1) and 80.6% (95% CI 75.5–85.7), respectively.

**Figure 3:**
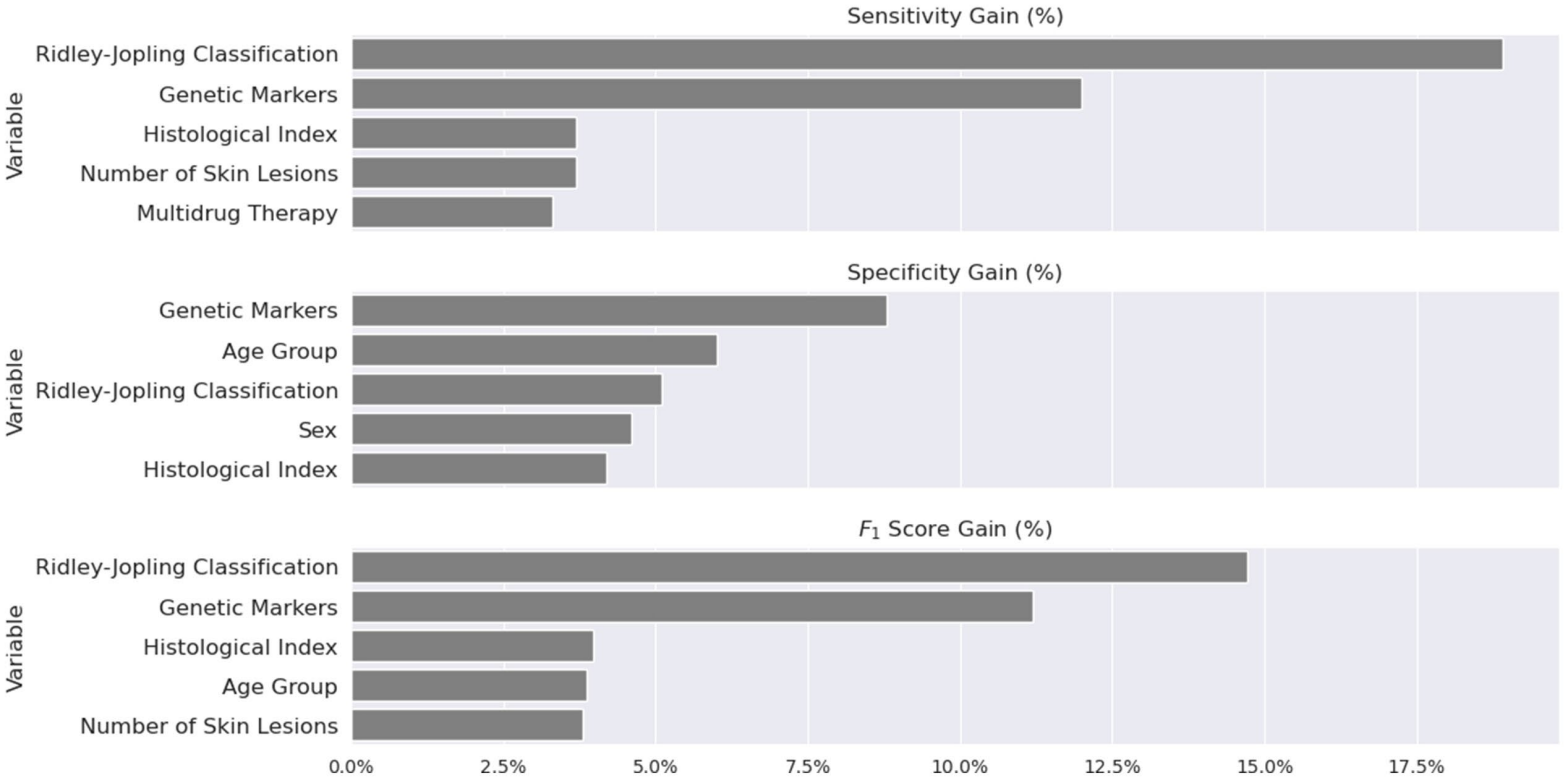
Top 5 most essential features measured in relative gain using sensitivity, specificity, and the F1 score.

To assess the importance of each of the variables individually, modeling was carried out after removing one at a time, and the impact on system performance was measured through changes in sensitivity, specificity, and F1. As summarized in Figure 3, the three attributes exerting the highest impact were R&J classification, combined genetic markers, and histological index. Interestingly, the highest estimates of accuracy, sensitivity, specificity, and both negative and positive predictive values were observed for the Bauru and the Goiania datasets, for which genotypic data was available, even higher than what was observed for the combined dataset of much larger sample size (the only exception being the positive predictive value for Bauru: 82.7% vs. 85.1% for the combined dataset) (Table 3).

**Table 3:** Results obtained for each population sample.

Population sample	Two-by-two contingency				Results	95% CI
Combined		NRLP	LR	Total	Sensitivity = 79.3%	73.9–84.7%
	NRLP	187	45	232	Specificity = 86.2%	81.6–90.8%
	LR	30	172	202	PVP = 85.1%	80.2–90.1%
	Total	217	217	434	PVN = 80.6%	75.5–85.7%
					Accuracy = 82.7%	79.2–86.3%
Goiania		NRLP	LR	Total	Sensitivity = 85.7%	76.5–94.9%
	NRLP	59	8	67	Specificity = 89.4%	82.0–96.8%
	LR	7	48	55	PVP = 87.3%	78.5–96.1%
	Total	66	56	122	PVN = 88.0%	80.3–95.8%
					Accuracy = 87.7%	81.9–93.5%
Bauru		NRLP	LR	Total	Sensitivity = 82.7%	72.4–93.0%
	NRLP	51	9	60	Specificity = 85.0%	76.0–94.0%
	LR	9	43	52	PVP = 82.7%	72.4–93.0%
	Total	60	52	112	PVN = 85.0%	76.0–94.0%
					Accuracy = 83.9%	77.1–90.7%
Fortaleza		NRLP	LR	Total	Sensitivity = 78.1%	68.6–87.6%
	NRLP	62	16	78	Specificity = 71.3%	61.8–80.8%
	LR	25	57	82	PVP = 69.5%	59.5–79.5%
	Total	87	73	160	PVN = 79.4%	70.5–88.4%
					Accuracy = 74.3%	67.6–81.1%
Manaus		NRLP	LR	Total	Sensitivity = 77.8%	58.6–97.0%
	NRLP	18	4	22	Specificity = 78.3%	61.4–95.1%
	LR	5	14	19	PVP = 73.7%	53.9–93.5%
	Total	23	18	41	PVN = 81.8%	65.7–97.9%
					Accuracy = 78.0%	65.4–90.7%

LR, leprosy reactions; NRLP, non-reactional leprosy patients; PPV, positive predictive value; NPV, negative predictive value; CI, confidence interval.

## 4. Discussion

As an outcome of contact with its causative agent, leprosy is controlled by multiple environmental and socioeconomic factors and innate characteristics of both the host and pathogen. The specific contribution of each of these factors to the risk of developing leprosy and its endophenotypes is widely unknown. Today, LRs constitute a significant cause of disabilities associated with leprosy; thus, predicting patients at higher risk of developing LR at the time of leprosy diagnosis may help prevent permanent neural impairment. However, an accurate estimate of this risk demands analyzing a very complex set of variables, which is difficult – if not impossible – to perform by an unassisted primary healthcare professional. Here we present an easy-to-use, flexible, and automated system that identifies leprosy patients at increased risk of developing LR based on clinical, socio-economical, laboratory, and genetic data. Patients at high risk are candidates for close monitoring during and after treatment, aiming to prompt the management of these aggressive events, minimizing the likelihood of permanent disabilities. Our platform translates basic scientific data into a direct application that may immediately impact leprosy patients’ quality of life and leprosy control programs’ effectiveness.

The three features that exerted the highest impact on the system’s performance were the R&J classification, the histological index, and the combined effect of the genetic markers (Figure 3). The R&J class is a well-accepted major risk factor for reversal reactions (7, 13, 21, 22, 37, 40, 41). As expected, simulations confirm that patients in the tuberculoid pole of the spectrum tend to have a higher chance of developing no reversal reaction (98% ~ when the classification is TT). As clinical form moves towards borderline, the probability of a T1R rises from <1 to 53%~ when the category is BB and, finally, patients at the lepromatous pole have a higher risk of developing T2R – more specifically, 61%~ when the type is LL. The second top-three parameter impacting the system is the histological index. An index equal to 2+ increases the risk of T1R to 56%~; values higher than 5+ shift the risk towards T2R – 45%~ when the histological index is 6+. This behavior is expected since an increase in the histological index is highly correlated with a higher bacterial load and, consequently, a move toward the lepromatous pole of the disease. A histological index higher than 5+ is also a well-known risk factor for developing T2R (38, 39). Finally, genetic data seems critical to improving the system’s performance, which suggests that understanding the true, exact nature of LR depends on the description of the underlying genetic mechanisms.

We are aware of the study’s limitations: we have had limited access to genetic information across the population samples; including genotypic data for additional, known LR susceptibility genes would likely positively impact the system’s performance. In addition, the heterogeneity of the databases, originally obtained for independent studies of distinct designs, prevented a comprehensive analysis of the performance of the system, which we understand was yet quite remarkable, likely due to the ability of Bayesian methods to estimate risk using all available – even if partial – information. This is important considering that not all leprosy centers across the globe will have access to molecular data of all the patients; in these cases, the platform can still help estimate the risk of LR using only the clinical/laboratory and demographic data with fair sensitivity and specificity, as observed for the Fortaleza and Manaus datasets (Table 3). Of note: the heterogeneity of the dataset is known to enhance the quality of a trained model, since it tends to improve the generalization capturing a more comprehensive understanding of the problem and its nuances. Thus, the inclusion of diverse datasets is a known strategy to improve the performance of machine learning models. For example, in the field of Random Forests, the use of diverse datasets has been explored as a method to enhance the model’s accuracy and robustness (78). This principle extends to various domains, including computer vision (79), and conversational AI (80). For a comprehensive evaluation and refining of the system, datasets enrolled prospectively with these specific purposes will be necessary.

## 5. Conclusion

We produced SEPAREH as an easy-to-use, online, free-access system that identifies leprosy patients at higher risk of developing LR. We believe that SEPAREH can be useful to help primary healthcare services to establish a protocol for patient follow-up dedicated to improving early diagnosis and prevention of the devastating consequences of untreated LR. Ultimately, risk assessment of LR for individual patients may be of potential positive impact on the prevention of permanent disabilities, the quality of life of the patients, and upon leprosy control programs.

## Data availability statement

The raw data supporting the conclusions of this article will be made available by the authors, without undue reservation.

## Ethics statement

This study was approved by the Brazilian Committee for Ethics in Research (CONEP) (protocol 1.722.447). All patients signed an informed consent to participate in the study; for patients <18 years old, the informed consent was signed by one of the parents or the legal guardian.

## Author contributions

RA, EH, DC, CM, and JN contributed to defining the AI-based protocol and data modeling. LH, GR, and EH developed the online platform. MA contributed to the recruitment and clinical characterization of the Tropical Pathology and Public Health Institute, Goiania, Goiás patients. AL, CS, AB, and PR contributed to the recruitment and clinical description of the Lauro de Souza Lima Institute, Bauru, São Paulo patients. MP and HS contributed to the recruitment and clinical characterization of the Dona Libânia Dermatology Centre patients, Fortaleza, Ceará. RC contributed to the recruitment and clinical description of the Alfredo da Matta Foundation patients, Manaus, Amazonas. MO contributed to the statistical analysis. VF contributed to the generation of the original genetic data. SB-S, GP, and MP contributed to coordinating the original study under which the population samples were recruited and characterized. RA, EH, CM, JN, and MM helped to draft the manuscript. MM is the principal investigator, the main responsible for the study design and execution, and provided senior supervision throughout the study. All authors contributed to the article and approved the submitted version.

## Funding

This work was supported by the Araucaria Foundation (Grant #41617.433.32610.10092013), the Brazilian Coordenação de Aperfeiçoamento de Pessoal de Nível Superior (CAPES), and the Leprosy Research Initiative (LRI)/Turing Foundation, grant ID# 704.16.31. MM is a Conselho Nacional de Desenvolvimento Científico e Tecnológico (CNPq) productivity (PQ) researcher level 2, grant #304368/2018-0. MA is under a research fellowship grant from the Brazilian Research Council/CNPq (Grant #311986-2019-6).

## Conflict of interest

The authors declare that the research was conducted in the absence of any commercial or financial relationships that could be construed as a potential conflict of interest.

## Publisher’s note

All claims expressed in this article are solely those of the authors and do not necessarily represent those of their affiliated organizations, or those of the publisher, the editors and the reviewers. Any product that may be evaluated in this article, or claim that may be made by its manufacturer, is not guaranteed or endorsed by the publisher.

## Footnotes

^1^The access to the platform is limited to HTTPS protocol. In case of difficulty accessing the platform, please certify whether HTTPS is being used.
