# Supplementary material for: Prediction of the occurrence of leprosy reactions based on Bayesian networks
Source: Front Med (Lausanne). 2023 Jul 26;10:1233220. doi: 10.3389/fmed.2023.1233220 (PMC10411956; doi:10.3389/fmed.2023.1233220)
Supplement: Supplementary file 1 [file Table_1.DOCX]

**Supplementary Table 1: Parameter definition for each of the selected variables included in the system**

| **Table S1 – Parameter definition for each of the selected variables included in the system** | | | | | | | |
| --- | --- | --- | --- | --- | --- | --- | --- |
| **Sex** | Male | Female |  |  |  |  |  |
| **Age Group** | 0 to 19 years | 20 to 39 years | 40 to 64 years | Elderly |  |  |  |
| **Multidrug Therapy** | PB - 6 Months | MB - 12 Months |  |  |  |  |  |
| **Ethnicity** | White | Black | Mixed Race | Yellow |  |  |  |
| **Ridley-Jopling Classification** | TT | BT | BB | BL | LL | Indeterminate |  |
| **First Signs and Symptoms (Self-Report)** | 0 to 1 | 1 to 2 | 2 to 3 | 3+ |  |  |  |
| **First Degree** | Yes | No |  |  |  |  |  |
| **Second Degree** | Yes | No |  |  |  |  |  |
| **Contact** | Yes | No |  |  |  |  |  |
| **Number of Skin Lesions** | 1 to 5 | 6 to 11 | Diffuse Infiltration |  |  |  |  |
| **Type of Lesion** | Plaques | Papules | Macules | Nodules | Diffuse Infiltration |  |  |
| **Color of Lesion** | Normochromic | Hypochromic | Hyperchromic | Erythematous |  |  |  |
| **Sensibility Testing** | Normal | Impairment | Dubious |  |  |  |  |
| **Bacilloscopic Index** | 1+ | 2+ | 3+ | 4+ | 5+ | 6+ | Negative |
| **Histological Index** | 1+ | 2+ | 3+ | 4+ | 5+ | 6+ | Negative |
| **PGL-1** | 1+ | 2+ | 3+ | 4+ | Negative |  |  |
| **Gene *IL6* - rs2069832** | AA + AG | GG |  |  |  |  |  |
| **Gene *IL6* - rs2069840** | CC | GG + CG |  |  |  |  |  |
| **Gene *IL6* - rs2069845** | AA | GG + AG |  |  |  |  |  |
| **Gene *IL6* - rs1800795** | CC + CG | GG |  |  |  |  |  |
| **Gene *TNFSF8* - rs6478108** | AA | AG | GG |  |  |  |  |
| **Gene *TNFSF8* - rs7863183** | CC | CT | TT |  |  |  |  |
| **Gene *TNFSF8* - rs1555457** | AA | AG | GG |  |  |  |  |
| **Gene *TNFSF8* - rs3181348** | AA | AG | GG |  |  |  |  |
| **Gene *ENSG00000235140* - rs7090170** | AA | AG | GG |  |  |  |  |
| **Gene *ENSG00000235140* - rs10826321** | CT | TT |  |  |  |  |  |
| **Gene *ENSG00000235140* - rs1875147** | CC | CT | TT |  |  |  |  |
| **Gene *ENSG00000235140* - rs7916086** | AA | AG | GG |  |  |  |  |
| **Gene *LRRK2* - rs4768236** | AA | CC |  |  |  |  |  |
| **Gene *LRRK2* - rs3761863** | AA | AG | GG |  |  |  |  |
| **Gene *LRRK2* - rs3886747** | CC | CT | TT |  |  |  |  |
| **Gene *NOD2* - rs8057341** | AA | AG | GG |  |  |  |  |
| **Gene *TLR1* I60S2 - rs5743618** | GG | GT | TT |  |  |  |  |
| **Gene *TLR1* N248S - rs4833095** | AA | AG | GG |  |  |  |  |

**Supplementary Table 2 –** Distribution of the parameters included in the system across the four databases

|  | | | | | | |
| --- | --- | --- | --- | --- | --- | --- |
| **Goiania** |  | **Manaus** |  | **Fortaleza** |  | **Bauru** |
| Sex |  | Sex |  | Sex |  | Sex |
| Age Group |  | Age Group |  | Age Group |  | Age Group |
| Ridley-Jopling Classification |  | Ridley-Jopling Classification |  | Ridley-Jopling Classification |  | Ridley-Jopling Classification |
| Multidrug Therapy |  | Multidrug Therapy |  | Multidrug Therapy |  | Multidrug Therapy |
| First Signs and Symptoms^a^ |  | First Signs and Symptoms^a^ |  | First Signs and Symptoms^a^ |  |  |
|  |  | Ethnicity |  | Ethnicity |  | Ethnicity |
|  |  | Bacilloscopic Index |  | Bacilloscopic Index |  | Bacilloscopic Index |
|  |  | Sensibility Testing |  | Sensibility Testing |  |  |
|  |  | Number of Skin Lesions |  | Number of Skin Lesions |  |  |
|  |  | Type of Lesion |  | Type of Lesion |  |  |
|  |  | Color of Lesion |  | Color of Lesion |  |  |
|  |  | PGL-1 |  | PGL-1 |  |  |
| Family History^b,c and d^ |  |  |  |  |  | Family History^b,c and d^ |
|  |  |  |  |  |  | Histological Index |
|  |  |  |  |  |  | *NOD2* - rs8057341 |
|  |  |  |  |  |  | *TLR1* N248S - rs4833095 |
|  |  |  |  |  |  | *TLR1* I60S2 - rs5743618 |
| *IL6* - rs2069832 |  |  |  |  |  |  |
| *IL6* - rs2069840 |  |  |  |  |  |  |
| *IL6* - rs2069845 |  |  |  |  |  |  |
| *IL6* - rs1800795 |  |  |  |  |  |  |
| *TNFSF8* - rs6478108 |  |  |  |  |  |  |
| *TNFSF8* - rs7863183 |  |  |  |  |  |  |
| *TNFSF8* - rs1555457 |  |  |  |  |  |  |
| *TNFSF8* - rs3181348 |  |  |  |  |  |  |
| *ENSG00000235140* - rs7090170 |  |  |  |  |  |  |
| *ENSG00000235140* - rs10826321 |  |  |  |  |  |  |
| *ENSG00000235140* - rs1875147 |  |  |  |  |  |  |
| *ENSG00000235140* - rs7916086 |  |  |  |  |  |  |
| *LRRK2* - rs4768236 |  |  |  |  |  |  |
| *LRRK2* - rs3761863 |  |  |  |  |  |  |
| *LRRK2* - rs3886747 |  |  |  |  |  |  |

^a^ Self-Report in years of the early signs and symptoms of leprosy.

^b^ Father, mother, child and sibs affected by leprosy.

^c^ Cousins, nephews, uncles/aunts, grandparents and grandchildren affected by leprosy.

^d^ Close contact affected by leprosy.
